# School-based harm reduction with adolescents: a pilot study

**DOI:** 10.1186/s13011-022-00502-1

**Published:** 2022-12-12

**Authors:** Nina Rose Fischer

**Affiliations:** John Jay College of Criminal Justice, 524 W. 59th Street Rm. 6.65.09, New York, NY 91001 USA

**Keywords:** Harm reduction education, Classroom based substance use curriculum, Adolescent substance use, Mixed methods research

## Abstract

A pilot study of Safety First: Real Drug Education for Teens showed significant results pre to post curriculum with high school freshmen. Negative outcomes of drug education are linked to a failure to engage students because of developmentally inappropriate materials that include activities that have no relevance to real experiences of young people. The few harm reduction studies showed increased student drug related knowledge. Students were less likely to consume substances, and less likely to consume to harmful levels. More studies are necessary to evidence harm reduction efficacy in the classroom. The goal of this study was to measure harm reduction knowledge and behaviors, including drug policy advocacy, before and after Safety First. Data were analyzed using McNemar’s test, ANOVA, linear regression, *t*-tests and thematic coding. Survey results, corroborated by the qualitative findings, showed a significant increase (*p* < .05) in high school freshmen harm reduction knowledge and behaviors in relationship to substance use pre to post Safety First. This increase related to a decrease in overall substance use. Harm reduction is often perceived as a controversial approach to substance use. These findings have implications for further study of what could be a promising harm reduction-based substance use intervention with teens.

**Supplementary Information:**

The online version contains supplementary material available at 10.1186/s13011-022-00502-1.

Research has shown that common reasons drug education programs for youth have failed were lack of student interest because they were not developmentally appropriate, or because activities did not relate to their actual lives [1, 2]. A review of school-based drug education studies [1] showed that for substance use education programs to be effective they should be based on the real experiences of young people, a harm reduction principle [1–3]. The study of Drug Policy Alliance’s (DPA) Safety First: Real Drug Education for Teens (hyperlinked) drug education curriculum for health education classes is grounded in harm reduction theory. The objective of the curriculum is to teach substance use harm reduction to support positive outcomes for young people.

## Background

### Harm reduction theory

Harm reduction theory includes pragmatic strategies aimed at reducing dangers related to substance use. The theory emerged with the discovery of AIDS in 1981. Harm reduction was important for reducing transmission of blood-borne infections and for addressing drug use. Evidence has shown that harm reduction approaches greatly reduce morbidity and mortality associated with risky substance use behaviors [4–6] but has rarely been used to inform drug education curriculum for teenagers.

Harm reduction is an ecological systems approach, addressing drug use from the micro level, individuals, families and communities to the macro level, local, state, and federal policies and norms [3, 7–9]. The theory promotes social justice with an emphasis on users’ rights, health, social and economic development, as opposed to the demonization of drug consumption [10]. Critical to the practice of harm reduction is recognizing that realities of poverty, class, racism, social isolation, past trauma, sex and gender-based discrimination and other social inequalities affect people’s capacity to address drug-related harm. Aims of this study were to measure student ability to understand and advocate for socially just harm reduction policy pre and post Safety First.

Harm reduction interventions vary according to dynamic needs of individuals and communities. The goals are to meet substance users “where they’re at,” incorporating a spectrum of strategies from abstinence, to managing use, to addressing conditions of use along with use itself. The theory adopts tenets of the trans theoretical stages of change model [11, 12] and motivational counseling [13]. This non-judgmental, amoral approach encourages people to embark on incremental, harm-reducing goals. A harm reduction approach is congruent with what is known about adolescent development and decision-making. However, the most prevalent drug education for teens has been abstinence based, attaching stigma and moral judgment to substance use and users, instead of learning the effects and how to make informed, healthy decisions about use [14, 15].

School based harm reduction programs have rarely received the attention of researchers. Limited studies exist about harm reduction drug education with adolescents in the US [1]. Only a few studies, from Canada, Australia and the UK showed positive results [1, 2, 16, 17]. Classroom based harm reduction approaches are limited but are gaining traction in school settings because of the mixed or ineffective results from prevention and abstinence-based programs that failed to meet the real needs of youth [2, 18]. The small pool of studies showed increase in drug related knowledge. Students were less likely to consume substances and were less likely to consume to harmful levels with themselves and peers [1, 2, 16, 17]. Harm reduction can potentially address the shortfalls of prevention programs but remains contentious in the context of youth substance use, thus has not been widely studied within this population [2].

Dr. Marsha Rosenbaum, the founder of Drug Policy Alliance (DPA) developed a pamphlet for parents about harm reduction and teens in 1999 where she defined principles for school drug education and ultimately for the Safety First curriculum, “Parents and teachers are responsible for engaging students, providing them with credible information [to] make responsible decisions, avoid drug abuse, and stay safe. Curricula should be age-specific, emphasize student participation, and provide science-based educational materials.” Harm reduction principles require a non-judgmental, motivational, culturally relevant, actively engaging environment that puts student experience at the center of the curriculum [2]. Safety first includes these elements.

Safety First teaches students about different types of drugs including the short and long-term effects. Students learn how to identify viable research about drugs and discuss and present their findings in the classroom. Drug beliefs are discussed, myths are dispelled, and facts are validated. Behaviors associated with substance use are studied and discussed to inform student’s future decision making. These key principles make up the operational definition of harm reduction reflected in the Safety First curriculum and measured in the study.

The Safety First curriculum developers trained teachers that participated in the pilot studies for three, 8 hours sessions and coached them weekly for at least an hour in the content and modalities of the curriculum. The developers provided technical assistance for curriculum implementation. All teachers delivered the curriculum one to two times per week, depending on the schedule of their health classes, in each of the schools. The class lasted one semester, up to 14 sessions, at 55 minutes per class. The materials necessary for each class were all easily accessible through free downloads online and physically from the DPA curriculum developer/trainers. “How the curriculum was taught” was the variable that had the most effect on the efficacy of the curriculum and is analyzed below.

The overall goal of the study was to measure harm reduction knowledge and behaviors before and after Safety First. Diverse urban public schools were the foci for the pilots in New York City and San Francisco. Outcomes showed change from pre to post Safety First (*p* < .05) in knowledge and behaviors related to substance use. The results corroborated the findings from the few other similar studies [1, 2, 16, 17, 19]. This study evidenced need for further implementation of harm reduction based substance use curriculum as part of health education in high schools and for more research to measure the effects of the curriculum with various populations and locales.

### Hypotheses

The hypotheses of this study were related to the aims of the Safety First: Real Drug Education for Teens curriculum. The curriculum developers hoped to educate freshmen high school students about harm reduction knowledge and behavior. Students will 1) Acquire critical thinking skills to access and evaluate information about alcohol and other drugs [knowledge and behavior]; 2) Understand decision-making and goal setting skills that help students make healthy choices related to substance use [knowledge and behavior]; 3) Develop personal and social strategies to manage the risks, benefits and harms of alcohol and other drug use [behavior]; 4) Know the impact of drug policies on personal and community health [knowledge]; and 5) Learn to advocate for health-oriented drug policies [behaviors]. Thus student knowledge and behavior related to substance use and harm reduction were measured before and after Safety First as part of required health education classes to determine the efficacy of the curriculum.

## Methods

### Data collection

Hypotheses were tested through the collection of data from validated pre/post quantitative surveys (Additional file 1: Appendix A in the data portal: Appendices A-D can be found in the Data Portal linked here) with items that measured substance use and harm reduction knowledge and behaviors [20–22] pre/post qualitative focus groups and one on one interviews with semi-structured field-tested guides; and field observation, on a weekly basis in each class with a field tested template. The 14-session (55 minutes/class) curriculum was implemented and studied in four freshmen health education classes at a public school in New York City and five public schools, four classes each, in San Francisco, CA. Researchers committed to different class periods and conducted field observation on different class days weekly to ensure inter-rater reliability [23].

### Demographics (Table 1)

#### Participants

Students were recruited through both purposive and random sampling methods. Drug Police Alliance (DPA) built purposeful relationships with health teachers that wanted to implement Safety First as part of their required substance use unit in New York City. Relationships were built between DPA and San Francisco health teachers through the Adolescent Health Group- a Department of Education arm that oversaw health education curriculum. Students that participated in the pre/post focus groups and interviews were chosen randomly by alternating names on the class rosters.

**Table 1:** Socio-demographic characteristics of student participants

No Prior Drug Education	New York		San Francisco	
	*n*	*%*	*n*	*%*
	77	95.1	589	88
Totals (*n* = 666)				
Total 701				
Age	New York		San Francisco	
	*n*	*%*	*n*	*%*
13			6	1
14	33	40.2	425	68.8
15	48	58.5	171	27.7
16	1	.1	6	1.0
17	1	.1	8	1.3
18			1	.2
19			1	.2
Totals (*n* = 701)				
Gender	New York		San Francisco	
	*n*	*%*	*n*	*%*
Male	34	42	347	56.5
Female	44	54.3	267	43.5
Non-Binary	5	3.7	0	0
Total 701				
Race/Ethnicity	New York		San Francisco	
	*n*	*%*	*n*	*%*
1-Black	7	9	52	8.7
2-Latinx	16	19	125	20.9
3-Native			2	.3
4-Asian	16	19	280	46.7
5-Middle Eastern			12	2.0
6-White	36	43	51	8.5
7-Other	8	10	77	12.9
(included mixed race Black/Asian, White/Latinx, South Asian & Middle Eastern)				
Totals (*n* = 676)				
Total 701				
Sexuality			San Francisco	
(added to data collection for demographics based on initial pilot in NYC)				
			*n*	*%*
1-Straight			523	84.6
2-Gay			1	.2
3-Bisexual			38	6.1
4-Undecided			52	8.4
5-Lesbian			4	.6
Totals (*n* = 618)				
Total 701				
Youth Locale	New York		San Francisco	
	*n*	*%*	*n*	*%*
Brooklyn	31	37.8		
Manhattan	25	30.5		
Bronx	12	14.6		
Queens	13	15.9		
Visitacion Valley			152	25
Excelsior			143	23
Richmond, Fillmore & Laurel Hts.			141	23
Central Richmond & Outer Sunset			96	15
Mission			86	14
Totals (*n* = 699)				
Total 701				
Police Contact and Suspension	New York		San Francisco	
	*n*	*%*	*n*	*%*
Arrests or Stops	9	11	37	6
Suspension	4	4.9	13	2
Totals (*n* = 63)				
Total 701				
Grades	New York		San Francisco	
	*n*	*%*	*n*	*%*
1-A’s	55	66	313	0.6
2-B’s	28	34	167	27.0
3-C’s			96	15.5
4-D’s			22	.3.6
5-F’s			20	3.2
Totals (*n* = 701)				
Total 701				
Future Plans	New York		San Francisco	
	*n*	*%*	*n*	*%*
1-College	0	100	381	61.8
2-Graduate School	83	100	137	22.2
3-Just finish high school			79	12.8
4-May not finish high school			13	.2.1
5-Vocational school			7	1.1
Totals (*n* = 700)				
Total 700				
Religion			San Francisco	
(added to data collection for demographics based on findings from NYC pilot)				
			*n*	*%*
1-Muslim			14	2.5
2-Christian			204	36.0
3-Buddhist			47	8.3
4-Jewish			13	2.3
5-Other/Agnostic/Atheist			289	51.0
Totals (*n* = 567)				
Total 701				

The total number of freshmen surveyed in the overall pool was 701. Some students did not answer demographic questions which accounted for reduced “*n*” (Table 1). The items “What is the definition of abstinence” and “What is the definition of harm reduction” write in examples, were added to the San Francisco survey based on the findings from the initial New York City study. Thus the “*n*” for those items is less. Prior to Safety First most students had not received any drug education (96%). Students were 14 (62%) and 15 years old (31%). Outliers included 13, 16, 17, 18 & 19 years old (7%). Students were males (54%), and female (45.6%). In New York City two identified as “Other” and one as gender non-conforming (0.4%). The largest total ethnic/racial group was Asian (43%), then Latinx (22%), mixed race (12%), white (12%), Black (9%), Middle Eastern (1.8%) and Native American (.02%). In New York City white students were the largest ethnic/racial group, however youth of color made up the majority of the student population. In San Francisco Asian students were the majority student population, then Latinx. Black and white students were next with the same representation. Most New York City students resided in Brooklyn and Manhattan while other students were closely split between Queens and the Bronx. Most San Francisco students lived in Visitacion Valley and Excelsior district. Central Richmond, Outer Sunset and the Mission district vied for second. A small number of students in both cities reported police contact, arrest and/or suspension (Table 1). Youth reported substance use as a reason for police involvement.

### Sample comparability

The total sample included three higher and three lower achieving schools, all public. The New York City school was unique because students applied and interviewed to be accepted. Pupils were high achieving coming in, average grades were “A’s” and “B’s.” All students planned to attend college and graduate school. Two out of the five San Francisco public schools were like the New York City site in grades and graduation rates but were not admissions based. The remaining three schools had students with lower grade point averages, with more of a range when asked about future plans. All were in politically progressive US coastal cities. All were ethnically diverse, and to an extent reflective of their city’s populations. All schools consisted of students from diverse economic backgrounds. Thus, this body of research from a sample of 701 students in New York City and San Francisco could possibly be extrapolated to students in similar locales with diverse achievement levels, racial and class demographics (Table 1).

### Data analysis

McNemar’s test was applied to analyze if the harm reduction knowledge and behavior change from before to after Safety First was significant on four critical items (Table 2). One-way ANOVA tests were conducted to determine if there was an effect by demographics on substance use knowledge and behavior survey responses (Additional file 1: Appendix B-D). Linear regression was employed to determine if race or gender were predictive of responses. Qualitative responses were aggregated using thematic codes based on the emergent themes from the “write in” responses on the pre/post surveys, and the interview and focus group transcription and were transformed into quantitative codes to count and compare student responses (Table 2 below, and items 40–44 in Additional file 1: Appendix A and Appendix B in data portal). Outcomes showed that students learned critical thinking, decision-making and harm reduction strategies. Items that did not show remarkable results, or were null, also informed future implications for Safety First.

**Table 2:** Changes in knowledge before and after safety first

	% Correct Before	% Correct After	McNemar Exact *p* <
What is harm reduction?	35.6	80.3	0.001
What is abstinence?	25.9	62.0	0.001
How to Detect an Opioid Overdose?	1.0	41.7	0.001
How would you advocate for a drug policy?	2.6	52.0	0.001

Note: correct responses: what is harm reduction? Reduce harms related to substance use thru i.e., dose and dosage, set and setting; testing contents. What is abstinence? Abstaining from substance use; describe how to identify an overdose on opioids. CUPS - cold, and clammy, unresponsiveness, puking, and sweating; how would you advocate for a drug policy? Harm reduction related activities i.e., restorative justice interventions like counseling and education in lieu of suspension, expulsion or arrest

## Results

The purpose of this pilot study was to determine if DPA’s newly rolled out Safety First: Real Drug education for Teens potentially increased harm reduction knowledge and behaviors for high school freshmen. The findings from the pre and post survey, fortified by the qualitative data, showed a likely increase in student harm reduction knowledge about drug contents and effects, drug research, positive behaviors related to substance use, and drug policies. The results demonstrated that the curriculum most likely influenced overall student substance use knowledge and behavior.

Students showed change in knowledge about, and behaviors related to harm reduction, abstinence, how to detect an opioid overdose, school specific drug policies, and how to advocate for harm reduction based drug policy after Safety First (*p* < .001) (Table 2). Students were more involved with advocacy activities after Safety First than before (*p* < .001). It is likely that learning about activism and advocacy as part of the curriculum contributed to this increase in advocacy activities (*p* < .001). More youth advocated for less punitive drug policies after Safety First (*p* < .001).

Themes about drug policy advocacy that emerged from the qualitative data collected from the students after the class pointed to “creating systems of support,” “reducing stigma,” and “lessening punishments.” When before Safety First the themes were advocacy for suspension and jail time. Students mentioned passing along what they learned to fellow classmates, family members, and school administrators after the class to help them improve decision-making about drugs and create fairer drug policies.

ANOVA tests revealed that the most influential effect on student response was from the school they attended, indicating that how a specific teacher taught the curriculum most likely mattered (see below and Appendices B-D). Students from specific schools post Safety First showed more understanding of drug policies, how to advocate for harm reduction based initiatives, and how to respond to an opioid overdose (Table 2). However, there was remarkable change across all student comprehension despite differences in how the curriculum was taught.

### Likert scale pre to post

Paired *t-*tests were conducted to determine if there was a significant difference between students’ scores on 20 Likert Scale items after the drug education course. The scale was one strongly agree and five strongly disagree. Seventeen were significant from pre to post Safety First (*p* < .001) (Additional file 1: Appendix C). Two of the three items that had no statistical significance, “People do not become dependent upon marijuana,” and “If you overdose on a drug you will die,” still showed a shift towards disagree, the harm reduction response, through means comparison. The item “It is better not to drink water while using MDMA (“molly”)” did not show a significant change. The students agreed more with this statement after Safety First. The harm reduction answer was strongly disagree. More students also agreed that “Alcohol helps you deal with uncomfortable feelings” which showed a significant change from pre to post (*p* < .037), producing a null hypothesis. This outcome provides valuable feedback to the Safety First developers. They need to review how Safety First addresses harm reduction related to MDMA and alcohol.

### Gender and race

For San Francisco, an Independent Sample *t*-test showed “Gender” mattered on two items. More males strongly disagreed that “Marijuana is safe because it is all natural,” than females (*p* < .001). More females moved to strongly agreeing that “You can die from drinking too much alcohol at one time” after Safety First than males (*p* < .001). An independent *t*-test was administered to measure if gender had an impact on students’ scores on the Likert Scale items. There was a significant difference between males and females on two items in New York City (Additional file 1: Appendix C). Females were less likely to agree than males that, “People do not become dependent on marijuana,” (*p* < .05). Females were also less likely than males to agree that zero tolerance drug policies make schools safer (*p* < .05). A linear regression demonstrated that race and gender (*p* > .05) were not predictive of significantly different test scores in either city. In San Francisco more males strongly disagreed than females about the item “Marijuana is safe because it is all natural” (*p* < .001). On the item “You can die from drinking too much alcohol at one time” females more strongly agreed than males (*p* < .001).

An ANOVA test showed that race and religion had an effect on student responses. Asian students were more likely to move towards disagreeing with the statement “Marijuana is safe because it is all natural” which was the harm reduction response, in comparison to Latinx and Black students (*p* < .001). Muslim students were more likely to move towards disagreeing with the statement “People do not become dependent upon marijuana,” in comparison to Jewish students (*p* = .020). ANOVA tests showed school site had the most influence on student responses to the Likert Scale items from pre to post (Additional file 1: Appendix C).

### Pre to post: substance use behaviors

On the pre/post survey there were questions about amount and likelihood of specific substance use: 1) to understand prevalence of substance use amongst the population; and 2) to see if learning about harm reduction influenced students’ behaviors/decision making. The majority of students did not report smoking or vaping tobacco but the few students that did, smoked a significant amount, this did not change from pre to post. For marijuana, students reported decreased use from pre to post (*p* < .001) (see below and Additional file 1: Appendix D). Marijuana use with a date showed remarkable change from “I would probably not use” to almost completely “I would definitely not use marijuana” (*p* < .001). There was a decrease in alcohol use from pre to post (*p* < .001). There was also an overall decrease in students reporting prescription drug use (*p* < .001) (Additional file 1: Appendix D).

ANOVA tests were administered to see if the demographic factors had an effect on the substance use behavior outcomes from pre to post Safety First (Additional file 1: Appendix D). A one-way AVOVA yielded that Asian students were more likely to move towards “I would definitely not take/smoke weed with family” than Black students (*p* = .002). An independent sample *t*-test evidenced that young men were more likely than young women to use prescription drugs with friends (*p* = .020). Results evidenced that students learned about harm reduction strategies. Prevalence of substance use amongst the population became clearer; harm reduction influenced students’ substance use behaviors/decision making from pre to post especially in relationship to marijuana and prescription drugs (Additional file 1: Appendix D).

More students believed that their classmates were using substances after Safety First than before. This change indicated that the class could have made the students more aware of substance use prevalence. This reported prevalence reflected national numbers for this age group [24]. In 2016 SAMSHA’s comprehensive report on drug abuse and health showed that 7.3 million youth between 12 and 20 reported alcohol use. About 1 in 5 drank alcohol in the past month. An estimated 855,000 adolescents aged 12 to 17 smoked cigarettes in the past month [24]. An approximated 24.0 million 12 or older in 2016 were current users of marijuana and approximately 1.6 million adolescents used marijuana in the past month. The national study spoke to the prevalence of drug use by 14- and 15-year-old young people shown in the study [24]. Student receptivity to harm reduction strategies, substantiated collaterally through the overall reduction in student use, validated the potential relevance of this approach with high school students, starting with freshmen.

### Overall harm reduction knowledge and behavior change

Thematic qualitative coding was used to identify the most emergent themes in this data. A code was assigned to prevalent themes and counted and compared to determine outcomes (Additional file 1: Appendix B). Young people demonstrated an understanding of key harm reduction thought processes and strategies solidifying successful aspects of the Safety First curriculum [3]. Students made change in their ability to describe specific harm reduction strategies possibly due to Safety First (*p* < .001). In response to “What would you do to make substance use safer?” More youth responded “1” “Realize and plan for set/setting and limits around goal setting related to substance use,” or understand the “Contents, dose, and dosage” than narrowly, “reduce harm” [3] after the class (Additional file 1: Appendix B).

### Neighborhood, class and race

Interviews unearthed themes related to a difference in student perceptions about substances based on neighborhood, class and race. Students that lived in lower income neighborhoods that were predominantly black and brown consistently believed that one should not do drugs because of the consequences observed in the community. For example, when asked, “What happens in your community when someone is under the influence of drugs or is found with drugs on them?” A 14-year-old African American young woman from Brownsville Brooklyn responded in the pre and post interview, “Arrest. People get shot. People go to the hospital. People go to jail.”

When asked the same question before the class, a white female student that lived in the Upper Westside of Manhattan stated,

I have to admit that I live in a privileged neighborhood. So the use of drugs actually wouldn’t be that bad. Because it’s not like there’s the strongest police force patrolling my neighborhood, which is a huge part of it, like a part that I have to admit.

When asked the same question after Safety First she answered, “… there’s such a low risk for me to be put in a position where I’m...criminalized. So I don’t have to worry walking down the street if I have weed with me or something.”

When asked, “Are different groups of people treated differently if they have or are using drugs? If so, how?” the same African American young woman above explained the neighborhood, class and race differences:

If you seem like a person from a rich up town neighborhood or family using them [drugs], you would immediately think that they got them from somebody else. And then you will look to someone from a poor community who has them [drugs] and blame them, which is a stereotype that I really hate. I think that most of the times if someone from a rich family gets caught with drugs, they’re not gonna get nothing more than a warning. If someone from a poor community or an African or the Hispanic race gets caught, they are going to jail.

A young white woman from an affluent neighborhood’s pre response corroborated her response through her answer to the same question,

At my middle school there was a situation where a guy, mixed race black and white, bought weed for his friend, a white girl. Then she was high in school with that weed. She didn’t even get into as much trouble as the kid who bought it. Everyone in the school was pointing out, he’s biracial, so he’s black. He had a two-week out of school suspension for buying her the weed off campus and she had nothing.

Her post response to the question, “Are different groups of people treated differently if they have or are using drugs? If so, how?” was informed by the drug policy race and class session,

For sure. Low-income groups, African American communities, people of color in general, are so much quicker to be criminalized and prosecuted for having drugs, especially marijuana. I know now that there’s a disproportionate incarceration rate for men of color caught with marijuana.

Themes from student interviews, focus groups, and “write in” answers about the unequal treatment of people using or selling substances because of race, class and neighborhood reflected class lessons from Safety First about inequality in drug policy implementation. The findings indicated that the class increased student knowledge about critical social justice topics. Social justice is key to the harm reduction approach [25].

### Student evaluation of safety first

The majority of students had a positive evaluation of Safety First. Fifty-five percent (*n* = 389) of students reported that they would recommend Safety First. Thirty-nine percent (*n* = 274) stated they would recommend Safety First with some changes. Six percent (*n* = 45) relayed they would not recommend Safety First. Thus 94% of the students believed Safety First was a worthwhile experience. Quantitative coding of the most prevalent themes from the qualitative data sources informed what the students liked best about Safety First.

Direct quotes exemplified the coded themes: Code “1” learning about harm reduction strategies, including what to do in an overdose, a non-judgmental approach to teaching drug education, and I liked ‘everything’: “I actually learned a lot and didn’t feel like I was just being told that drugs were awful, and trying them makes you an awful person,” “I learned how to be safe and smart;” “High schoolers are more prepared for anything involving drug usage and overdose;” “It was not one of those ‘DARE’ abstinence only curriculums where they try to convince you that weed is a gateway to heroine and you will die if you try molly. I actually felt like I learned something that wasn’t fear based;” and “You seem to have tried really hard to make this curriculum great and it shows.” Code “2” learning about different substances: “I like learning about the different effects different drugs can do to your brain and body.” Code “3” the interactive/engaging activities and liking how the teacher taught the class overall, “I liked the different activities that we did that demonstrated different scenarios and substances, also the teacher explained it very well” and “I liked the part where we drank the Koolaid for a party experiment.” Code “4” videos and mixed media, “The videos including the ASAP science videos,” and “I absolutely love that youtube channel,” “I liked the videos, they were informative.” Code “5” was “Nothing” or “I Don’t Know.” “Learning about specific substances” (*n* = 216, 40%) was what the majority of students liked about Safety First. Students wrote “Nothing” or Didn’t Know second (*n* = 137, 25%); the interactive and engaging activities third (*n* = 87, 16%); learning harm reduction strategies fourth (*n* = 81, 15%) and videos were the least mentioned (*n* = 18, 3.3%).

“No Judgement,” “Harm Reduction Skills,” and “Real Drug Education” were other themes that emerged in the post evaluation of the curriculum: “I liked that it wasn’t very judgmental and understood that the chance of kids trying drugs is likely. I also liked the harm reduction strategies,” “I liked how the curriculum went in depth about the side effects of drugs and taught us how to research and find correct information about a drug. It was well organized, and I got so much out of it,” and “It did not look down on people who used! Safety First stated facts and was looking out for our well beings; no biased opinions.”

The data illustrated that youth learned about both harm reduction skills and knowledge, appreciated the non-judgmental element of the approach and enjoyed when it was taught using dynamic, interactive teaching modalities with mixed media.

## Discussion

The results demonstrated that after Safety First student harm reduction knowledge and behavior changed after Safety First (*p* < .05). Prevalence of substance use amongst this student population became clearer. The issue of prevalence, as described above, is quite critical. Regardless of their moral beliefs parents, teachers, administrators, policy makers and a continuum of social services need to know that 14- and 15-year old’s are using substances, and for some, a remarkable amount daily and weekly (see below and Additional file 1: Appendix D). Entrenched beliefs by policy makers and institutions that “abstinence-based drug education is more effective” persist even with the preponderance of evidence to expose their inefficacy and actual harm [14, 15].

The goals of the Safety First developers did not expressly include reducing substance use. True harm reduction does not stigmatize substance use or assume that it is inevitably “wrong” or “dangerous.” [3] As a researcher I was curious about whether there would be a collateral effect from the curriculum on student drug use, since institutions that promote drug education often see reduced use and abstinence as a goal. Collateral findings did show a significant relationship (*p* < .05) between increased knowledge and skills with reduced substance use over the course of the semester. Teaching students harm reduction influenced students’ substance use behaviors/decision making from pre to post especially in relationship to marijuana and prescription drugs (below and Additional file 1: Appendix D).

### Likert scale items

Seventeen of the Likert scale items on the pre/post survey were significant from pre to post Safety First because students’ answers demonstrated an increase in harm reduction knowledge and behaviors (*p* < .001) (Additional file 1: Appendix C). The item “It is better not to drink water while using MDMA (“molly”)” did not show a significant change. The students agreed more with this statement after Safety First. The harm reduction answer was to “strongly disagree.” More students also agreed that “Alcohol helps you deal with uncomfortable feelings” which showed a significant change from pre to post (*p* = .037), producing a null hypothesis. The harm reduction answer was to “strongly disagree.” This outcome provided valuable feedback to the Safety First developers. They need to review how Safety First addresses harm reduction related to MDMA and alcohol.

### The teaching effect

ANOVA tests revealed that the most influential effect on student knowledge and behavior change was from the school they attended. How the curriculum was taught was the most influential variable. Teachers need training and coaching about how to implement Safety First. Technical assistance must be available from the purveyor or other trained experts to ensure fidelity. Importantly, there was still remarkable change across all student comprehension despite differences in how the curriculum was taught.

### Study limitations with recommendations

The recommendations that stem from the “Discussion” are to include more curricula about MDMA and alcohol; provide coaching, training and technical assistance for teachers to adhere to fidelity of Safety First and to use dynamic, interactive, engaging pedagogical modalities in the classroom.

### Abundance of data

An abundance of data points were collected for this study. More explication and discussion of fidelity issues, classroom observations and teacher evaluations are rich fodder for future manuscripts. Further discussion and recommendations could be mined from additional analysis. An article that dives more deeply into solely the qualitative data would give nuanced texture to the unique narrative of the Safety First classroom experience. Ethnography and phenomenology could both be used for the data analysis of interviews, focus groups, field observations and “write in” survey data to produce additional, compelling literature.

### Sustainability

Although there have been no longitudinal studies of a high school substance use harm reduction curriculum, research of drug prevention programs over time showed that positive effects last throughout high school but taper off after [26]. Most schools only require one semester of health. This pilot study showed that in 14 classes students learned advocacy skills to promote creative harm reduction oriented policies. A sustainability recommendation is for drug policy organizations to spearhead advocacy groups on school campuses so students can sustain the harm reduction messages throughout and after high school. Longitudinal studies to measure student behavior and knowledge over time are key to the sustainability of Safety First.

### Transportability

Results from public schools in two urban coastal cities showed a remarkable change from pre to post Safety First. This study tested student response across literacy, class and achievement levels. The study population were an integrated, multicultural cohort of 14- and 15-year old’s in urban areas, and these discrete demographic groups- Asian (296), Latinx (141), male (381) and female (311) exceeded 100. A sample must be over 100 to be considered generalizable [27]. Thus, in order to expand the transportability of the results it is integral to see how Safety First works in suburban, rural or small predominantly white locales; or with predominantly Black youth in smaller towns or large cities [23]. Lesbian, Bisexual, Trans, Non-Binary and Gay youth should be study participants. Youth in “last chance” schools, on probation, in detention or elite private schools should also be identified. Can Safety First be implemented successfully in a different type of institution? A drug treatment facility or a community-based organization? Does the curriculum work with middle school youth or older teens/young adults? Future research should serve youth of different ages, across similar and new demographic factors, and in environments outside the purview of this study.

### Randomized control groups

The scope and scale of this study did not allow for the randomized control groups. These would have allowed a direct comparison of the outcomes for young people that either did not have a substance use component in their health class or had been exposed to a prevention and/or abstinence-based curriculum. Future studies should include randomized control groups across various populations of youth. Albeit, this pre/post study design did show baseline student knowledge and behaviors and the effects of Safety first on students after the curriculum.

## Conclusion

The Safety First: Real Drug Education for Teens curriculum had significant effect on a diverse population of freshmen from six public high schools in the United States. Students acquired critical thinking skills to access and evaluate information about alcohol and other drugs; they had a better understanding of decision-making and goal setting skills that increased healthy choices related to substance use; they developed personal and social strategies to manage the risks, benefits and harms of alcohol and other drug use; they knew the impact of drug policies on personal and community health; and students learned to advocate for health-oriented drug policies. Outcomes inform future research. The implications of the results were that Safety First should be tested at comparable and new school sites. Further study should include randomized survey samples and control groups. The generalizability of the results should be measured with similar and different populations, as well as test the same students overtime to show the endurance of the effects.

The results are timely. Student knowledge increase related to the detection and response to an opioid overdose is particularly relevant because of national prevalence [28]. Student interviews about unequal treatment of people using or selling drugs based on race, class, gender and neighborhood illustrated the importance of understanding the intersection particularly between drug policy, race and class. There are a dearth of studies about harm reduction in the classroom [2, 16, 19]. These pilot findings are seed for future research to support harm reduction education for youth.

### Supplementary Information

**Additional file 1.** .

## Data Availability

Data is included in the Tables below and Additional Tables and Appendices accessible through this link to DropBox.

## Appendix

### Summary Pre and Post Substance Use Behaviors

Tobacco use showed no significant change form pre to post. On average, youth reported being with youth that used tobacco or that they used tobacco themselves monthly or never (3.70) before and after Safety First. On average, youth reported being with youth that used alcohol, or using alcohol themselves monthly or never (3.70) before and after Safety First. Tobacco and alcohol showed no significant change from pre to post. Marijuana was a different story. Students believed that fewer peers used marijuana on average (31%) after Safety First than before the harm reduction unit (43%). Students reported spending more time with students that used marijuana on average from monthly or never (Mean-μ = 3.29) closer to monthly (μ = 3.15). Youth reported marijuana use was monthly or never (μ = 3.80) pre to post.

Marijuana use showed a significant change from “I would probably not use” to almost completely “I would definitely not use” if “...your date is using marijuana” after Safety First. Prescription drug use and alcohol use showed no significant change from pre to post, staying an average between “I would probably not use” to “I would definitely not use.”

Students made a remarkable change from pre to post in their ability to describe specific harm reduction strategies in response to “What would you do to make substance use safer?**”** Average youth response moved from “2” just reduce harm (μ = 2.25) to “1” Realize and plan for set/setting and limits around goal setting related to substance use, or Contents, Dose, Dosage including reduction of use (μ = 1.60).

An ANOVA was administered to see if any of the demographic factors had an effect on the substance use behavior outcomes from pre to post Safety First. Race and gender had the only effects. A one-way AVOVA yielded that Asian students were more likely to move towards “I would definitely not take/smoke weed with family” than black students [F(6, 556) = 3.50, *p* = .002]. An independent sample *t*-test evidenced that young men were more likely than young women to use prescription drugs with friends (Mean-μ = −.92) to (μ = − 1.31), t(111) = 2.35, *p* = .020.

The above results evidenced that the curriculum taught the students about harm reduction strategies. Prevalence of substance use amongst the population became more clear; harm reduction seemed to influence students’ substance use behaviors/decision making from pre to post Safety First, especially in relationship to marijuana and prescription drugs; and students clearly demonstrated an increase in knowledge of harm reduction strategies.
