# Supplementary material for: School-based harm reduction with adolescents: a pilot study
Source: Subst Abuse Treat Prev Policy. 2022 Dec 12;17:79. doi: 10.1186/s13011-022-00502-1 (PMC9743577; doi:10.1186/s13011-022-00502-1)
Supplement: Supplementary file 1 — Additional file 1. . [file 13011_2022_502_MOESM1_ESM.docx]

**APPENDICES**

**Table of Contents**

**Appendix A- Pre/Post Survey**……………………………………………………………..1-5

**Appendix B- Student Evaluation by Demographic Data**………………………………..5-13

**Appendix C- Likert Scale Findings**………………………………………………………13-20

**Appendix D- Pre and Post Substance Use Behaviors**……………………………………20-26

**APPENDIX A**

*Safety First: Real Drug Education for Teens* Pre-Class Youth Survey

**Class Location/Date:**

**Please tell us about yourself:**

| How old are you? ________ | What is your race/ethnicity? Check all that apply. |
| --- | --- |
| What is your gender? Male Female Other______ | Black / African American  White |
| What neighborhood do you live in?______________________ | Hispanic / Latino Asian  Native American  Other________________ |

**1) During the past three months have you… (Please circle either YES or NO)**

**2) Participated in any other programs focused on substance use** *(using substances, such as alcohol or drugs)***?**

YES NO

Please describe:

**3) Gotten into a fight with other youth at school or around the neighborhood?** YES NO Please describe:

**4) Been arrested or stopped by the police?** YES NO

**If YES, for what?**

**5) Been suspended?** YES NO **If YES, for what?**

**6) Chosen to volunteer to help out or stand up for a cause?** YES

**Questions about drugs and drug policy:**

**7)** **Describe how to identify an overdose on opioids** (*A class of drugs that relieve pain, examples include heroin, fentanyl, oxycodone, and morphine*).

**8) What is the drug policy** *(rules and regulations regarding drugs or drug use)* **at your school?**

**9) How would you advocate for a drug policy? At your school? And in your community?**

| 1) The definition of “drug” includes sugar and caffeine. |
| --- |
| 2) Reviewing one online source about a substance is enough to know its effects. |
| 3) You should call 911 if someone is overdosing. |
| 4) Using alcohol can cause dependence and addiction. |
| 5) Marijuana is safe because it is all natural. |
| 6) People do not become dependent upon marijuana. |
| 7) If someone drinks too much alcohol, vomits and passes out, you should let them sleep it off. |
| 8) You can die from drinking too much alcohol at one time. |
| 9) Alcohol helps you deal with uncomfortable feelings. |
| 10) Marijuana edibles take effect immediately. |
| 11) Zero tolerance drug policies make schools safer. |
| 12) Oxycontin is less addictive than heroin. |
| 13) A single injection of heroin can cause an overdose. |
| 14) It is better not to drink water while using MDMA (“molly”). |
| 15) If you overdose on a drug you will die. |
| 16) Crack and cocaine have different active ingredients. |
| 17) It is safe to use someone else’s prescription drug if you both have the same symptoms. |
| 18) It is safe to take Adderall and other prescription stimulants to stay awake and study. |
| 19) Where someone uses a drug, or, setting, contributes to how safe their use is. |
| 20) Crack is more dangerous than cocaine. |

**21) What grades do you get in school? (Mark one)**

☐ Mostly A’s ☐ Mostly B’s ☐ Mostly C’s ☐ Mostly D’s ☐ Mostly F’s

**22) What is the highest level of school you plan to finish? (Mark one)**

☐ I may not finish high school ☐ I plan to finish high school ☐ I plan to go to vocational or trade school after high school graduation ☐ I plan to go to college ☐ I plan to go to graduate school or professional school (law, medicine)

**23) How often are you with youth who are smoking cigarettes? (Mark one)**

**☐** Daily ☐ Weekly ☐ Monthly ☐ Never

**24) On the days you smoke cigarettes, how many do you usually smoke? (Mark one)**

☐ Less than 1 cigarette a day ☐ 1 or 2 cigarettes a day ☐ 3 to 7 cigarettes a day ☐ About 1/2 pack of cigarettes a day ☐ A pack or more of cigarettes a day ☐ I don’t smoke cigarettes

**25) About what percent (%) of students in your grade do you think smoked cigarettes one or more times in the last month?** Your best guess is fine. _______ %

**26) How often are you with youth who are drinking alcohol? (Mark one)**

**☐** Daily ☐ Weekly ☐ Monthly ☐ Never

**27) About what percent (%) of students in your grade do you think drank alcohol one or more times in the last month? By alcohol we mean beer, wine or hard liquor.** Your best guess is fine. _______ %

**28) On how many days did you have any alcohol in the last month? (Mark one**)

☐ None ☐ 1 or 2 days in the last month ☐ 3 to 5 days in the last month ☐ 6 to 19 **days** in the last month ☐ 20 or more days in the last month

**29) Who usually offers you alcohol? (Mark one)**

☐ My friends ☐ Kids I don’t know well ☐ My brother or sister ☐ Adults I know well ☐ Adults I don’t know well ☐ No one offers me alcohol

**30) About what percent (%) of the students in your grade do you think used marijuana one or more times in the last month? Your best guess is fine**. _______ %

**31) On how many days did you use any marijuana in the last month? (Mark one)**

☐ None ☐ 1 or 2 days in the last month ☐ 3 to 5 days in the last month ☐ 6 to 19 days in the last month ☐ 20 or more days in the last month

32) **How often are you with youth who are smoking marijuana? (Mark one)**

**☐** Daily ☐ Weekly ☐ Monthly ☐ Never

**33)** **On the days you use marijuana, about how many times do you use it? (Mark one)**

☐ Once a day ☐ Twice a day ☐ 3 or more times a day ☐ I don’t use marijuana

**34) Suppose you are offered marijuana. What would you do in this situation? (Mark one box for each item)**

**Suppose:** a. **Your best friend is using marijuana**

☐ I would definitely use marijuana ☐ I would probably use marijuana ☐ I would probably not use marijuana ☐ I would definitely not use marijuana

**Suppose:** **b. Your date is using marijuana**

☐ I would definitely use marijuana ☐ I would probably use marijuana ☐ I would probably not use marijuana ☐ I would definitely not use marijuana

**Suppose:** **c. A family member offers you marijuana**

☐ I would definitely use marijuana ☐ I would probably use marijuana ☐ I would probably not use marijuana ☐ I would definitely not use marijuana

**Suppose: d. You’re at a party where everyone is using it.**

☐ I would definitely use marijuana ☐ I would probably use marijuana ☐ I would probably not use marijuana ☐ I would definitely not use marijuana

**35)** **Suppose you are offered alcohol. What would you do in this situation? (Mark one box for each item)**

**Suppose:** **a. Your best friend is using alcohol**

☐ I would definitely use alcohol ☐ I would probably use alcohol ☐ I would probably not use alcohol ☐ I would definitely not use alcohol

**Suppose:** **b. Your date is using alcohol**

☐ I would definitely use alcohol ☐ I would probably use alcohol ☐ I would probably not use alcohol ☐ I would definitely not use alcohol

**Suppose: c. A family member offers you alcohol**

☐ I would definitely use alcohol ☐ I would probably use alcohol ☐ I would probably not use alcohol ☐ I would definitely not use alcohol

**Suppose: d. At a party where everyone is using it.**

☐ I would definitely use alcohol ☐ I would probably use alcohol ☐ I would probably not use alcohol ☐ I would definitely not use alcohol

**36)** Suppose you are offered a prescription drug (e.g., Adderall, OxyContin, Vicodin, Valium, cough syrup) and you do not have a prescription:

**Suppose:** **a. Your best friend is using the prescription drug**

☐ I would definitely use the prescription drug ☐ I would probably use the prescription drug l ☐ I would probably not use the prescription drug ☐ I would definitely not use the prescription drug

**Suppose:** **b. Your date is using the prescription drug**

☐ I would definitely use the prescription drug ☐ I would probably use the prescription drug ☐ I would probably not use the prescription drug ☐ I would definitely not use the prescription drug

**Suppose:** **c. A family member offers you the prescription drug**

☐ I would definitely use the prescription drug ☐ I would probably use the prescription drug ☐ I would probably not use the prescription drug ☐ I would definitely not use the prescription drug

**Suppose: d. At a party where everyone is using it.**

☐ I would definitely use the prescription drug ☐ I would probably use the prescription drug ☐ I would probably not use the prescription drug ☐ I would definitely not use the prescription drug

POST SURVEY

**40)** **What did you think of the *Safety First: Real Drug Education for Teens curriculum?*** (**Mark one box**)

I would highly recommend it to other students ☐ I would recommend it with some changes ☐ I would not recommend it at all☐

If you marked “I would recommend it with some changes,” please describe what you would change:

If you marked, “I would not recommend it at all,” please explain why:

**41) What did you like best about the curriculum?**

**42) What did you like least about it?**

**43) What, if anything, would you change about the curriculum?**

**44) Name 3 harm reduction strategies that you will use for yourself, family or peers**.

**APPENDIX B**

Student Evaluation of Safety First

Students were asked the following questions to evaluate Safety First. Charts illustrate the responses.

**Would You Recommend Safety First?**

Students were asked on the post survey whether they would recommend Safety First. “1” was code for “I would highly recommend it,” “2” for “I would recommend with some changes” and

“3” for “I would not recommend it at all.” Fifty-five percent (n= 308) of students from five San Francisco Unified School District high schools reported that they would recommend Safety First. Thirty-seven percent (n =208) of the students stated they would recommend Safety First with some changes. Eight percent (n= 45) relayed they would not recommend Safety First. Thus 92% of the students believed Safety First was a useful experience.

**By School, Race, Religion, Grades, Future Plans and Gender**

A one-way ANOVA showed no effect from school, race, religion, grades or future plans on whether or not a student would recommend Safety First. An Independent t-test showed no significant relationship between recommending Safety First and gender.

**What did you Like Best about Safety First?**

What students liked best about Safety First was coded as “1” Learning about harm reduction strategies, including what to do in an overdose, a non judgmental approach to teaching drug education, and I liked “everything;” “2” learning about different substances; “3” the interactive/engaging activities including Kahoot, the Koolaid exercise and liking how the teacher taught the class overall; “4” Videos; and “5” Nothing or I Don’t Know. Student quotes best describe each code “1” through “5”:

1. *I actually learned a lot and didn’t feel like I was just being told that drugs were awful, and trying them makes you an awful person, but I learned how to be safe and smart.*

*High schoolers are more prepared for anything involving drug usage and overdose.*

*It was not one of those "DARE" abstinence only curriculums where they try to convince you that weed is a gateway to heroine and you will die if you try molly. I actually felt like I learned something that wasn't fear based. You seem to have tried really hard to make this curriculum great and it shows.*

1. *I like learning about the different effects different drugs can do to your brain and body*

*It was interesting to learn about the different drugs and what they do. Also the teacher explained it very well.*

1. I liked the different activities that we did that demonstrated different scenarios and substances etc.

I liked the part where we drank the Koolaid for a party experiment

1. *The salty kool-aid. Also, the ASAP science videos. I absolutely love that youtube channel*

*I liked the videos, they were informative*

1. *Nothing*

*Idk* [I don’t know]

“Learning about specific substances” (f= 216, 40%) was what the majority of students liked about Safety First. Students wrote “Nothing” or Didn’t Know second (f= 137, 25%); the interactive and engaging activities third (f= 87, 16%); learning harm reduction strategies fourth (f= 81, 15%) and videos were the least mentioned (f= 18, 3.3).

**By School, Race, Religion, Grades, Future Plans and Gender**

A one-way ANOVA showed no effect from school, race, religion, grades or future plans on what a student liked best about Safety First. An Independent t-test showed no significant relationship between what a student liked best about Safety First and gender.

**What would you change about Safety First?**

What students would change about Safety First was coded as “1” Less slides, packets and lectures. Make more engaging with hands on activities more fun and interactive. “2” More Information about different drugs, benefits, effects, historical context, more personal stories, treatment, and reasons for addiction to different substances; “3” Nothing; and “4” I don’t know.

Student quotes best described each code “1” through “4”:

1. *Less words, add more interesting things, and fun things on the slides.*

*More fun activities, field trips to hospitals, where drugs are processed, etc.*

*I would add more activities to make it more engaging.*

1. *I’d make sure that more information is provided on the specific types of drugs.*

*Include a larger variety of drugs.*

*I would add more information on the individual drugs instead of just the general category*

1. *Nothing.*

*I wouldn't change anything.*

*I think the program is fine how it is already.*

1. *IDK* [I don’t know]

“Nothing” (f= 238, 47%) was what the majority of students wanted to change about Safety First. Students wrote “I don’t know” second (f= 112, 22%); “Less slides, packets and lectures and more interactive and engaging activities” third (f= 91, 18%); and fourth “More Information...” (f= 68, 13%). Thus less than half of the students would not change anything about Safety First.

Thirty one percent would reduce the slides and packets and make the classes more interactive; and include more information about different drugs, benefits, effects, historical context, personal stories, treatment, and reasons for addiction to different substances. The quotes above give examples from students.

**By School, Race, Religion, Grades, Future Plans and Gender**

A one-way ANOVA showed no effect from school, race, religion, grades or future plans on what changes a student would make to Safety First. An Independent t-test showed no significant relationship between changes to Safety First and gender.

**Name Three Harm Reduction Strategies**

The harm reduction strategies were coded as “1” Realize and plan for set/setting and limits around goal setting; “2” Contents, dose, dosage including reduction of use; “3” Abstinence and alternatives to drugs; “4” Harm Reduction response to drug related emergencies like the recovery position; “5” I Don’t Know; and 6 “Advocacy.” Student quotes best described each code “1” through “6”:

1. *When you smoke weed do it after school or over the weekends not in school.*

*Be in a safe place with people you trust*

*Do it* [drugs at] *an appropriate time of the day and situation*

1. *Edibles juts eat it 1 and no more because you can overdose*

*Small doses*

*Drinking water with beer and molly*

1. *dont smoke dont smoke dont smoke*

*Drink water instead of alcohol*

*Exercise*

1. *Call 911 in event of overdose*

*know how to respond to an emergency*

*Naloxone CPR*

1. *Idk* [I don’t know]
2. *spread posters*

*spread facts*

*I can advocate for the dismissal of Reagan era war-on-drugs policies that are making the world unsafer, and making drugs more dangerous and making it more dangerous for people addicted to get safe drugs.*

“Abstinence and alternatives to drugs” (f= 506, 32.2%) was what the majority of students chose as a harm reduction strategy. “Realize and plan for set/setting and limits around goal setting” was second (f= 378, 24%); “Contents, dose, dosage including reduction of use” was third (f= 359, 23%); fourth was “I don’t know” (f= 262, 17%). Fifth was harm reduction response to drug related emergencies, like the recovery position (f= 54, 3.4%); and lastly, advocacy (f= 13, .8%).

**By School**

A one-way ANOVA showed that attending Washington high school had a significant effect on change in knowledge of harm reduction in comparison to Wallenberg [F(4, 517) = 3.38, p= .010]. Washington shifted more towards “1” Realize and plan for set/setting and limits around goal setting; “2” Contents, dose, dosage including reduction of use; and Wallenberg towards “3” Abstinence and alternatives to drugs and “4” Harm Reduction response to drug related emergencies like the recovery position.

**By Race, Religion, Grades, Future Plans and Gender**

A one-way ANOVA showed no effect from between race, religion, grades, future plans on knowledge of harm reduction. An Independent t-test showed no significant relationship between harm reduction knowledge and gender.

**Summary Student Evaluation of Safety First**

Overall students from five public high schools in San Francisco would recommend Safety First Real Drug Education for Youth. Fifty-five percent (n= 308) would recommend the curriculum and 37% (n =208) would recommend Safety First with some changes. Eight percent (n= 45) relayed they would not recommend Safety First. Thus 92% of the students believed Safety First was a useful experience. When asked what student liked best about Safety First they relayed “Learning about specific substances” (f= 216, 40%), “Nothing” or Didn’t Know (f= 137, 25%); the interactive and engaging activities (f= 87, 16%); learning harm reduction strategies (f= 81, 15%) and videos (f= 18, 3.3).

**What Would You Change about Safety First?**

When asked what the students would change about Safety First youth relayed, “Nothing” (f= 238, 47%); “I don’t know” (f= 112, 22%); “Less slides, packets and lectures and more interactive and engaging activities” (f= 91, 18%); and “More Information about different drugs, benefits, effects, historical context more personal stories, treatment and reasons for addiction” (f= 68, 13%). Thus just less than half of the students (47%) would not change anything about Safety First. Thirty one percent would reduce the slides and packets and make the classes more interactive; and include more information about different drugs, benefits, effects, historical context, personal stories, treatment, and reasons for addiction to different substances. The quotes above give examples from students.

**Name Three Harm Reduction Strategies**

“Abstinence and alternatives to drugs” (f= 506, 32.2%) was the most prevalent harm reduction strategy recorded. “Realize and plan for set/setting and limits around goal setting” was second (f= 378, 24%); “Contents, dose, dosage including reduction of use” was third (f= 359, 23%); fourth was “I don’t know” (f= 262, 17%). Fifth was harm reduction response to drug related emergencies, like the recovery position (f= 54, 3.4%); and lastly, advocacy (f= 13, .8%).

**By School, Race, Religion, Grades, Future Plans and Gender**

There was no relationship between school, race, gender, religion, grades, future plans and whether or not a student would recommend Safety First, what they liked best, or what they would change. An ANOVA did show a significant relationship between Washington and Wallenberg in relationship to name 3 harm reduction strategies. More Washington students named “Set and Setting” and “Dose and Dosage” while more Wallenberg students wrote “Abstinence and Alternatives to Drugs.”

The student recommendations provide critical information for how to improve Safety First to make it more effective for the target population

**APPENDIX C**

Likert Scale Pre and Post Results

An independent t-test was conducted to ascertain if there was a significant difference between students’ scores on the Likert Scale items 1-20 between the pre-test and the post-test for the four classes tested. The test was scored from one through five, with one meaning that the students strongly agreed with the statement and five meaning that students strongly disagreed with the statement. The paired t-test showed that 17 out of the 20 items were significant. Two of the three items that did not show significance still showed the mean response going in the desired direction. Change in the dependent variable, the students harm reduction knowledge and behavior, is most likely due to Safety First.

**1) The definition of “drug” includes sugar and caffeine.** More students agreed with this statement after Safety First (M= 2.1, SD= 1.15) than before (M= 3.16, SD= 1.20), t(558) = 14.44, *p* = .001). Desired change in the students’ harm reduction knowledge and behavior is most likely due to Safety First.

**2) Reviewing one online source about a substance is enough to know its effects.** More students disagreed with this statement after Safety First (M= 3.74, SD= 1.07) than before (M= 3.60, SD= 1.07), t(560) = -2.040, *p* = .042). Desired change in the students’ harm reduction knowledge and behavior is most likely due to Safety First.

**3) You should call 911 if someone is overdosing.** More students strongly agreed with this statement after Safety First (M= 1.45, SD= .69) than before (M= 1.64, SD= .87), t(552) = 3.90, *p* = .001). Desired change in the students’ harm reduction knowledge and behavior is most likely due to Safety First.

**4) Using alcohol can cause dependence and addiction.** More students strongly agreed with this statement after Safety First (M= 1.75, SD= .79) than before (M= 1.97, SD= .90), t(555) = 4.11, *p* = .001). Desired change in the students’ harm reduction knowledge and behavior is most likely due to Safety First.

**5) Marijuana is safe because it is all natural.** More students disagreed with this statement after Safety First (M= 3.86, SD= 1.12) than before (M= 3.58, SD= 1.07), t(550) = -4.21, *p* = .001). Desired change in the students’ harm reduction knowledge and behavior is most likely due to Safety First.

6) **People do not become dependent upon marijuana** did not show a significant change from pre to post that can be attributed to Safety First. However the mean showed that on average the students disagreed more with this statement after Safety First (M= 3.56, SD= 1.11) than before (M= 3.45, SD= .98), t(544) = -1.70, *p* = .090).

**7) If someone drinks too much alcohol, vomits and passes out, you should let them sleep it off.** More students disagreed with this statement after Safety First (M= 4.03, SD= 1.12) than before (M= 3.47, SD= 1.20), t(553) = -8.53, p = .001). Desired change in the students’ harm reduction knowledge and behavior is most likely due to Safety First.

**8) You can die from drinking too much alcohol at one time.** More students strongly agreed with this statement after Safety First (M= 1.79, SD= 1.29) than before (M= 2.15, SD= 1.05), t(556) = 5.05, *p* = .001). Desired change in the students’ harm reduction knowledge and behavior is most likely due to Safety First.

**9) Alcohol helps you deal with uncomfortable feelings.** More students agreed with this statement after Safety First (M= 2.91, SD= 1.123) than before (M= 3.05, SD= 1.126), t(558) = -2.08, *p* = .037). This shows that more content about the effects of alcohol is necessary in the Safety First unit.

**10) Marijuana edibles take effect immediately.** More students disagreed with this statement after Safety First (M= 3.42, SD= 1.23) than before (M= 3.22, SD= .89), t(552) = -3.08, *p* = .002). Desired change in the students’ harm reduction knowledge and behavior is most likely due to Safety First.

**11) Zero tolerance drug policies make schools safer.** More students disagreed with this statement after Safety First (M= 3.42, SD= 1.22) than before (M= 2.76, SD= 1.19), t(543) = -8.84, *p* = .001). Desired change in the students’ harm reduction knowledge and behavior is most likely due to Safety First.

**12) Oxycontin is less addictive than heroin.** More students disagreed with this statement after Safety First (M= 3.27, SD= .80) than before (M= 3.10, SD= .62), t(554) = -3.80, *p* = .001). Desired change in the students’ harm reduction knowledge and behavior is most likely due to Safety First.

**13) A single injection of heroin can cause an overdose.** More students agreed with this statement after Safety First (M= 2.71, SD= 1.02) than before (M= 2.84, SD= .81), t(550) = 2.47, *p* = .014). Desired change in the students’ harm reduction knowledge and behavior is most likely due to Safety First.

14) **It is better not to drink water while using MDMA (“molly”)** did not show a significant change from pre to post that can be attributed to Safety First. The mean showed that on average the students agreed more with this statement after Safety First (M= 2.94, SD= .77) than before (M= 3.00, SD= .59), t(488) = 1.54, *p* = .123). The correct answer was strongly disagree. It is possible that the curriculum needs to more effectively address harm reduction related to MDMA.

15) **If you overdose on a drug you will die** did not show a significant change from pre to post that can be attributed to Safety First. However, more students did disagree with this statement after Safety First (M= 2.90, SD= 1.23) than before (M= 2.78, SD= 1.10), t(543) = 1.70, *p* = .089).

**16) Crack and cocaine have different active ingredients.** More students disagreed with this statement after Safety First (M= 3.47, SD= 1.23) than before (M= 2.88, SD= .77), t(548) = -9.65, *p* = .001). Desired change in the students’ harm reduction knowledge and behavior is most likely due to Safety First.

**17) It is safe to use someone else’s prescription drug if you both have the same symptoms.** More students disagreed with this statement after Safety First (M= 4.18, SD= .97) than before (M= 3.82, SD= .99), t(551) = -6.12, *p* = .001). Desired change in the students’ harm reduction knowledge and behavior is most likely due to Safety First.

**18) It is safe to take Adderall and other prescription stimulants to stay awake and study.** More students disagreed with this statement after Safety First (M= 3.84, SD= .99) than before (M= 3.48, SD= .95), t(557) = -6.29, *p* = .001). Desired change in the students’ harm reduction knowledge and behavior is most likely due to Safety First.

**19) Where someone uses a drug, or, setting, contributes to how safe their use is.** More students agreed with this statement after Safety First (M= 2.54, SD= 1.20) than before (M= 3.17, SD= .88), t(553) = 9.75, *p* = .001). Desired change in the students’ harm reduction knowledge and behavior is most likely due to Safety First.

**20) Crack is more dangerous than cocaine.** More students disagreed with this statement after Safety First (M= 3.45, SD= 1.01) than before (M= 3.03, SD= .74), t(559) = -7.50, *p* = .001). Desired change in the students’ harm reduction knowledge and behavior is most likely due to Safety First.

**Gender and Likert Scale Items**

The t-tests demonstrated that students from all genders showed an average desired change from pre to post for most of the Likert scale items. I conducted an independent sample t-test to ascertain if gender had an impact on students’ scores on the Likert Scale items. There was a significant difference between males and females **on two** of the 20 Likert Scale items.

- Both males and females moved towards disagreeing that “**Marijuana is safe because it is all natural”** after Safety First**.** Males had a larger increase from (µ = 3.43) to (µ = 3.88), t(602) = -3.67, p =.001 and therefore on more strongly disagreed than females.
- Both males and females moved to strongly agreeing that “**You can die from drinking too much alcohol at one time”** after Safety First**.** Females had a larger increase than from (µ = 2.36) to (µ = 1.80), t(601) = -4.14, *p* =.001 and therefore more strongly agreed than males.

**Schools and Likert Scale Items**

The t-tests showed that overall students from every school showed an average desired change from pre to post for most of the Likert scale items. ANOVA tests answered if the school the students attended had a significant effect on their responses. The following items showed significant effect based on school placement.

- **The definition of “drug” includes sugar and caffeine:** A one-way AVOVA yielded that attending Washington high school had a significant effect on the desired student response [F(4, 553) = 12.95, *p* = .001]. Washington students were more likely to move towards agreeing with the statement than students from all the other schools.
- **Marijuana is safe because it is all natural:** A one-way AVOVA yielded that attending Wallenberg high school had a significant effect on the desired student response [F(4, 545) = 2.73, *p* = .028] in comparison to Mission. Students from Wallenberg were less likely to move towards agreeing with the statement than students from Mission.
- **Marijuana edibles take effect immediately:** A one-way AVOVA yielded that attending Mission in comparison to Burton and Wallenberg, and attending Washington and Balboa in comparison to Burton had a significant effect on the desired student response [F(4, 547) = 6.95, *p* = .001]. Students from Mission in comparison to Burton and Wallenberg, and Washington and Balboa students in comparison to Burton students were more likely to move towards disagreeing with the statement.
- **Zero tolerance drug policies make schools safer:** A one-way AVOVA yielded that attending Mission high school had a significant effect on the desired student response [F(4, 538) = 8.53, *p* = .001]. Mission students were more likely to shift towards disagreeing with the statement than the students from all the other schools.
- **Crack and cocaine have different active ingredients:** A one-way AVOVA yielded that attending Washington high school had a significant effect on the desired student response [F(4, 543) = 3.24, *p* = .012] in comparison to Burton. Students from Washington were more likely to shift towards disagreeing with the statement than students from Burton.
- **It is safe to use someone else’s prescription drug if you both have the same symptoms:** A one-way AVOVA yielded that attending Washington had a significant effect on the desired student response [F(4, 546) = 5.33, *p* = .001]. Students from Washington were more likely to shift towards disagreeing with the statement than students from Mission, Balboa and Burton.
- **Where someone uses a drug, or, setting, contributes to how safe their use is:** A one-way AVOVA yielded that attending Wallenberg high school had a significant effect on the desired student response [F(4, 546) = 5.33, *p* = .001]. Students from Wallenberg were more likely to move towards agreeing with the statement than students from Burton and Balboa.
- **Crack is more dangerous than cocaine:** A one-way AVOVA yielded that attending Wallenberg and Washington high schools had a significant effect on the desired student response [F(4, 546) = 5.33, *p* = .001]. Washington and Wallenberg were more likely to move towards disagreeing with the statement than students from Burton.

**Race and Likert Scale Items**

The t-tests showed that students from all races showed an average desired change from pre to post for most of the Likert scale items. ANOVA tests answered if the race/ethnicity of the students had a significant effect on their responses. The following items showed significant effect based on race/ethnicity.

- **Marijuana is safe because it is all natural:** A one-way AVOVA yielded that Asian students were more likely to move towards disagreeing with the statement, which was the desired student response, [F(6, 528) = 5.83, *p* = .001] in comparison to Latinx and Black students.
- **People do not become dependent upon marijuana:** A one-way AVOVA yielded that white students were more likely to move towards disagreeing with the statement, which was the desired student response, [F(6, 522) = 2.53, *p* = .020] in comparison to black students.
- **Where someone uses a drug, or, setting, contributes to how safe their use is:** A one-way AVOVA yielded that white students were more likely to move towards agreeing with the statement, which was the desired student response, [F(6, 522) = 2.53, *p* = .020] in comparison to Latinx students.

**Religion and Likert Scale Items**

The t-tests showed that students from all religions showed an average desired change from pre to post for most of the Likert scale items. ANOVA tests answered if the religion of the students had a significant effect on their responses. The following items showed significant effect based on religion.

- **People do not become dependent upon marijuana:** A one-way AVOVA yielded that Muslim students were more likely to move towards disagreeing with the statement, which was the desired student response, [F(4, 493) = 2.71, *p* = .029] in comparison to Jewish students.

**Summary**

Results of Likert Scale Pre and Post

I found that seventeen out of the 20 Likert Scale were significant from pre to post Safety First (p<0.001). Two out of the three items that had no statistical significance, “**People do not become dependent upon marijuana,**” and “**If you overdose on a drug you will die,**” still showed a shift towards disagree, the desired answer, through Cross Tabulation. The item “**It is better not to drink water while using MDMA (“molly”)**” did not show a significant change from pre to post that can be attributed to Safety First. The mean showed that on average the students agreed more with this statement after Safety First. The correct answer was strongly disagree. More students agreed with this statement after Safety First: “**Alcohol helps you deal with uncomfortable feelings.**” This shows that more content about the effects of alcohol is necessary in the Safety First unit. This outcome provides valuable feedback to the Safety First developers: It is possible that the curriculum needs to more effectively address harm reduction related to MDMA and alcohol.

An Independent Sample T-Test showed “Gender” mattered on two items. Males had a larger increase from pre to post on the item “**Marijuana is safe because it is all natural**,**”** than females. More males strongly disagreed with the statement. More females moved to strongly agreeing that “**You can die from drinking too much alcohol at one time”** after Safety First than males.

An ANOVA test showed that race mattered on three items and religion had an effect on one. The one showing the most effect by race was “**Marijuana is safe because it is all natural**.” Asian students were more likely to move towards disagreeing with the statement, which was the desired student response, in comparison to Latinx and Black students. Muslim students were more likely to move towards disagreeing with the statement “**People do not become dependent upon marijuana**,” which was the desired student response, in comparison to Jewish students.

AVOVA tests showed that school mattered most in relationship to the student responses on the Likert Scale items from pre to post. Student response varied on eight items based on school placement. These are some of the highlights. Washington students were more likely to move towards agreeing with the statement “**The definition of “drug” includes sugar and caffeine”** than students from all the other schools. Mission students were more likely to shift towards disagreeing with the statement “**Zero tolerance drug policies make schools safer**” than the students from all the other schools. Washington students were more likely to shift towards disagreeing with the statement “**It is safe to use someone else’s prescription drug if you both have the same symptoms**” than students from Mission, Balboa and Burton.

The outcomes from the Likert Scale items showed that overall students learned a significant amount of harm reduction knowledge from the Safety First curriculum.

**APPENDIX D**

Pre and Post Substance Use Behaviors

The curriculum taught the students about harm reduction strategies. On the pre/post survey there were questions about substance use behaviors: 1) to understand the prevalence of substance use amongst the population; 2) to see if learning about harm reduction influenced students’ substance use behaviors/decision making from pre to post Safety First; and 3) to investigate whether students learned harm reduction skills.

**Tobacco use**

The answers to “How often are you with youth who are smoking cigarettes?” “How often are you with youth who vape tobacco?” “If you smoke cigarettes, how many do you usually smoke?” and “How often do you vape tobacco?” were coded as “1” Daily, “2” Weekly,“3” Monthly, and “4” Never. A paired t-tests showed that all smoking and vaping tobacco behavior questions showed no significance across any of the items from pre to post. The average was about Monthly or Never for each item (3.70) pre and post. “About what percent (%) of students in your grade do you think smoked cigarettes or vaped tobacco one or more times in the last month?” also showed no significant change from pre to post. The average stayed the same for cigarettes (21%) and vaping tobacco (35%).

**Alcohol**

The answers to “How often are you with youth who are drinking alcohol?” “About what percent (%) of students in your grade do you think drank alcohol one or more times in the last month?” and “If you drink alcohol, how many days did you have any alcohol in the last month?” were coded as “1” Daily, “2” Weekly,“3” Monthly, and “4” Never. A paired t-tests showed that all alcohol questions showed no significance across any of the items from pre to post. The average was Monthly or Never for each item (3.70) pre and post. Youth believed on average 25% of their peers were drinking alcohol from pre to post Safety First.

**Marijuana Use**

The answers to “About what percent (%) of the students in your grade do you think used marijuana one or more times in the last month?” “On how many days did you use any marijuana in the last month?” “How often are you with youth who are using marijuana?” were coded as “1” Daily, “2” Weekly, “3” Monthly, and “4” Never. A paired t-test showed there was no significance from pre to post on any of the items about cigarettes, vaping or alcohol use. Marijuana use was a different story.

- Students perceptions of “**About what percentage of students in your grade used marijuana in the last month?**” changed significantly after Safety First (M= 31.25, SD= 29.01) in comparison to before (M= 42.45, SD= 69.96), t(551) = 3.46, *p* = .001). Students believed that fewer peers used marijuana on average (31%) after Safety First than before the harm reduction unit (43%). The t-test showed that this shift can be attributed to Safety First.
- “**How often are you with youth who are using marijuana?**” changed after Safety First (M= 3.15, SD= 1.11) in comparison to before (M= 3.29, SD= 1.20), t(586) = 1.96 *p* = .049). Students reported spending more time with students that used marijuana on average from monthly or never (3.29) closer to monthly (3.15). The *t*-test showed that this shift can be attributed to Safety First.
- “On how many days did you use any marijuana in the last month?” showed no significant change from pre to post. The average was monthly or never (3.80) pre to post.

**Summary**

Tobacco use showed no significant change form pre to post. On average, youth reported being with youth that used tobacco or that they used tobacco themselves monthly or never (Mean- µ = 3.70) before and after Safety First. Youth believed 21% of their peers were smoking tobacco one or more times in the past month. Youth believed 35% of their peers were vaping tobacco one or more times in the past month. On average, youth reported being with youth that used alcohol, or using alcohol themselves monthly or never (µ = 3.70) before and after Safety First. Youth believed 25% of their peers were drinking alcohol one or more times in the past month.

Tobacco and alcohol showed no significant change from pre to post. Marijuana was a different story. Students believed that fewer peers used marijuana on average (31%) after Safety First than before the harm reduction unit (43%). Students reported spending more time with students that used marijuana on average from monthly or never (µ = 3.29) closer to monthly (µ = 3.15). Youth reported marijuana use was monthly or never (µ = 3.80) pre to post.

**Would you use marijuana in the following scenarios?**

Students were asked, “Suppose you are offered marijuana. What would you do in this situation?”

The answers are coded as “1” I would definitely use marijuana “2” I would probably use marijuana “3” I would probably not use marijuana and “4” I would definitely not use marijuana. A paired t-test showed no significant change from pre to post in the students marijuana use except when using with a date.

- **Your best friend is using marijuana**. The average stayed around 3.32, I would probably not use to definitely not use marijuana, from pre to post.
- **Your date is using marijuana**. After Safety First, there was a significant change from pre to post in youth reporting they would definitely not use marijuana (M= 3.98, SD= 1.33) from probably not use marijuana (M= 3.43, SD= .873) if their date was using, t(581) = -8.22, *p* = .001). Average youth response moved from “I would probably not use” (3.43) to almost completely “I would definitely not use marijuana (3.98).
- **A family member offers you marijuana**. The average stayed around 3.39 I would probably not use to definitely not use marijuana, from pre to post.
- **You’re at a party where everyone is using marijuana**. The average stayed around 3.37 I would probably not use to definitely not use marijuana, from pre to post.

**Would you use alcohol in the following scenarios?**

Students were asked, “Suppose you are offered alcohol. What would you do in this situation?”

The answers were coded as “1” I would definitely use alcohol “2” I would probably use alcohol “3” I would probably not use alcohol and “4” I would definitely not use alcohol. A paired t-test showed **NO** significant change from pre to post in the students alcohol use in the following scenarios.

- **Your best friend is using alcohol**. The average stayed around 3.37, I would probably not use alcohol, from pre to post.
- **Your date is using alcohol**. The average stayed around 3.43, I would probably not use alcohol, from pre to post.
- **A family member offers you alcohol**. The average stayed around 3.30, I would probably not use alcohol, from pre to post.
- **You’re at a party where everyone is using alcohol**. The average stayed around 3.35, I would probably not use alcohol, from pre to post.

**Would you use prescription drugs in the following scenarios?**

Students were asked, “Suppose you are offered prescription drugs (e.g., Adderall, OxyContin, Vicodin, Valium, cough syrup). What would you do in this situation?” The answers were coded as “1” I would definitely use prescription drugs “2” I would probably use prescription drugs “3” I would probably not use prescription drugs and “4” I would definitely not use prescription drugs. A paired t-test showed **NO** significant change from pre to post in the students alcohol use in the following scenarios.

- **Your best friend is using prescription drugs**. The average stayed around 3.72, I would probably not use prescription drugs to I would definitely not use prescription drugs, from pre to post.
- **Your date is using prescription drugs**. The average stayed around 3.75, I would probably not use prescription drugs to I would definitely not use prescription drugs, from pre to post.
- **A family member offers you prescription drugs**. The average stayed around 3.63, I would probably not use prescription drugs to I would definitely not use prescription drugs, from pre to post.
- **You’re at a party where everyone is using prescription drugs**. The average stayed around 3.75, I would probably not use prescription drugs to I would definitely not use prescription drugs, from pre to post.

Student reported marijuana and prescription drug use in possible scenarios showed significant change from pre to post. Marijuana use showed a change from “I would probably not use” close to “I would probably not use.” Prescription drug use showed a more remarkable change from “I probably would use,” to “I would probably not use.” Alcohol showed no change from pre to post, staying at “I would probably not use.”

**What would you do to make substance use safer?**

Students were asked, “**What would you do to make substance use safer?”** Students wrote in their answers. I recoded them to reflect what was taught in Safety First: “1” Realize and plan for set/setting and limits around goal setting related to substance use, and/or Contents, Dose, Dosage including reduction of use, “2” just reduce harm; “3” just say no to drugs; and “4” I don’t know or incorrect.

- After Safety First, on average, students reported a specific harm reduction strategy learned in the course more often **(**M= 1.60, SD= .79) than before (M= 2.25, SD= .80), t(537) = 13.01, *p* = .001). Average youth response moved from “2” just reduce harm (µ = 2.25) to “1” Realize and plan for set/setting and limits around goal setting related to substance use, or Contents, Dose, Dosage including reduction of use (µ = 1.60).

Students made a remarkable change from pre to post in their ability to describe specific harm reduction strategies in response to “**What would you do to make substance use safer?”** Average youth response moved from “2” just reduce harm (µ = 2.25) to “1” Realize and plan for set/setting and limits around goal setting related to substance use, or Contents, Dose, Dosage including reduction of use (µ = 1.60).

Summary

Pre and Post Substance Use Behaviors

Tobacco use showed no significant change form pre to post. On average, youth reported being with youth that used tobacco or that they used tobacco themselves monthly or never (3.70) before and after Safety First. Youth believed 21% of their peers were smoking tobacco one or more times in the past month. Youth believed 35% of their peers were vaping tobacco one or more times in the past month. On average, youth reported being with youth that used alcohol, or using alcohol themselves monthly or never (3.70) before and after Safety First. Youth believed 25% of their peers were drinking alcohol one or more times in the past month.

Tobacco and alcohol showed no significant change from pre to post. Marijuana was a different story. Students believed that fewer peers used marijuana on average (31%) after Safety First than before the harm reduction unit (43%). Students reported spending more time with students that used marijuana on average from monthly or never (3.29) closer to monthly (3.15). Youth reported marijuana use was monthly or never (3.80) pre to post.

Marijuana use showed a significant change from “I would probably not use” to almost completely “I would definitely not use if “...your date is using marijuana” after Safety First. Prescription drug use and alcohol use showed no significant change from pre to post, staying an average between “I would probably not use to “I would definitely not use.”

Students made a remarkable change from pre to post in their ability to describe specific harm reduction strategies in response to “**What would you do to make substance use safer?”** Average youth response moved from “2” just reduce harm (2.25) to “1” Realize and plan for set/setting and limits around goal setting related to substance use, or Contents, Dose, Dosage including reduction of use (1.60).

An ANOVA was administered to see if any of the demographic factors had an effect on the substance use behavior outcomes from pre to post Safety First. Race and gender had the only effects. A one-way AVOVA yielded that Asian students were more likely to move towards “I would definitely not take/smoke weed with family” than black students [F(6, 556) = 3.50, *p* = .002]. An independent sample t-test evidenced that young men were more likely than young women to use prescription drugs with friends (µ = -.92) to (µ = -1.31), t(111) = 2.35, *p* =.020.

The above results evidence that the curriculum taught the students about harm reduction strategies. Prevalence of substance use amongst the population became more clear; harm reduction seemed to influence students’ substance use behaviors/decision making from pre to post Safety First, especially in relationship to marijuana and prescription drugs; and students clearly demonstrated an increase in knowledge of harm reduction strategies.
